# A methodological study revisiting obesity and lifestyle behaviors of Jordanian adolescents in Amman after 14 years

**DOI:** 10.3389/fspor.2026.1841128

**Published:** 2026-06-17

**Authors:** Hazzaa M. Al-Hazzaa, Adam T. Amawi, Loay M. Al-Shawabka, Fatima M. Alkhrissat, Omaya I. Dabayebeh, Rana M. Albaradi, Alia A. Alghwiri, Hasan A. Al-Saoud, Ibrahim M. Dabayebeh

**Affiliations:** 1 School of Sport Sciences, University of Jordan, Amman, Jordan; 2 Lifestyle and Health Research Center, Health Sciences Research Center, Princess Nourah Bint Abdulrahman University, Riyadh, Saudi Arabia; 3 Department of Movement Science and Training, School of Sport Sciences, University of Jordan, Amman, Jordan; 4 Department of Physical Education, School of Sport Sciences, University of Jordan, Amman, Jordan; 5 Al-Ahliyya Amman University, Amman, Jordan; 6 Ministry of Education and School of Sport Sciences, University of Jordan, Amman, Jordan; 7 Department of Physiotherapy, School of Rehabilitation Sciences, University of Jordan, Amman, Jordan

**Keywords:** adolescent, ATLS, dietary habit, obesity, physical activity, psychological health, sedentary behaviors, sleep

## Abstract

Lifestyle factors are associated with many adverse health outcomes. Adolescence represents a critical developmental period during which individual lifestyle behaviors are established. Regularly monitoring lifestyle behaviors, including physical activity (PA), sleep, dietary habits, and sedentary time, during adolescence is important. In 2011, a school-based study conducted in several major Arab cities including Amman, Jordan. The main findings indicated a considerable proportion of adolescents had unhealthy lifestyle behaviors and correlated with overweight/obesity. The present research aims to revisit the obesity levels, lifestyle behaviors and dietary habits of Jordanian adolescents (*N* = 1320) in Amman and examine the changing trend of these lifestyle behaviors after 14 years from the previous study. Weight, height, waist circumference, grip strength, psychological well-being will be measured. PA, sedentary behaviors, sleep, and dietary habits will be assessed using ATLS and ASBQ questionnaires. Making comparison between the past and the current studies provide evidence on the changes among Jordanian adolescents’ lifestyle behaviors over the course of more than a decade. The findings of this study will provide valuable data on overweight/obesity status and lifestyle behaviors of Jordanian youth. Such information can be used for planning and implementing health promotion programs for adolescents intended to reduce obesity and improve lifestyle behaviors.

## 1 Background

According to the world health organization (WHO), by the year 2030, non-communicable diseases (NCDs) are going to be the leading cause of death in every part of the world (1). The global NCD burden appears to be linked to a few modifiable behavioral risk factors (2). Indeed, physical inactivity and low intake of fruits and vegetables are considered by the WHO among the major yet preventable risk factors for NCDs (3). The detrimental effect of unhealthy lifestyle behaviors is well documented, as physical inactivity, sedentary behaviors, unhealthy dietary habits and insufficient sleep are shown to associate with many adverse health outcomes, like weight gain, obesity, reduced cardiorespiratory and musculoskeletal function, type 2 diabetes mellitus, insulin resistance, less favorable metabolic health, inflammation, decreased cognitive function, and negative psychological health (4–7). On the other hand, ample evidence has shown significant associations between healthy lifestyle and increased longevity, improved health, and disease prevention (4, 8, 9).

Adolescence represents a critical developmental period during which individual lifestyle behaviors are established (10). It is a period that can potentially impact later health and disease (11). Monitoring lifestyle behaviors, including physical activity and sedentary time, during adolescence is important. The Global Action Plan on Physical Activity 2018–2030 emphasizes the regular national surveillance of the population's physical activity (12). Recent research on tracking lifestyle habits showed that persistent physical inactivity from youth to adulthood was linked to increased risk of impaired glucose metabolism in adulthood (13). Further, a study conducted on Brazilian students showed that psychological well-being influences health behaviors differently in adolescent boys and girls (14). In addition, a recent systematic review indicated that a high level of physical activity and a low level of sedentary behavior were related to best physical health, psychological health, and education-related outcomes among children and adolescents (15). Despite the recognized benefits of regular physical activity, daily physical activity was shown to decrease during adolescence, and such a decrease is more marked among females (16). A recent report indicated that more than 80% of school-aged adolescents worldwide do not achieve the minimum level of recommended daily physical activity (17).

Previous research has revealed that childhood obesity is associated with many amendable lifestyle factors, including sedentary behaviors, physical inactivity and unhealthy dietary choices (18). Overweight and obesity amongst Jordanian adolescents (15–18-year-olds) using International Obesity Task Force (IOTF) Standards was reported to have recently reached 22.4% (19). Also, among Jordanian school children aged 7-18 years, physical activity and eating regular homemade meals were found to significantly reduce obesity (20). Moreover, findings from a study conducted on Chinese adolescents with high exercise-high calorie diet indicated the lowest risk of overweight and obesity, whereas adolescents with low physical activity and unhealthy exercise and dietary behaviors had the highest risk of overweight and obesity (21).

In 2011, as a part of the Arab Teens lifestyle Study (ATLS), a school-based, multicenter, cross-sectional study that was conducted in several major cities in the Arab countries (22–24), data were also collected from Jordanian adolescents in Amman (25, 26). The main findings showed that obesity prevalence was higher among male than female Jordanian adolescents; several lifestyle factors were related to overweight and obesity; and that a considerable proportion of adolescents had undesirable lifestyle behaviors. The present research project intends to revisit the obesity levels, lifestyle behaviors and dietary habits of Jordanian adolescents living in Amman city and examine the changing trend of these lifestyle behaviors after 14 years from the past study using the same research design, methodologies and instruments that were used in 2011. Additionally, the current research examines, for the first time, the psychological well-being among Jordanian adolescents and grip strength and relate these factors to lifestyle behaviors of Jordanian youth. Impaired psychological well-being was found in children and adolescents with severe obesity (27). Moreover, a high level of physical activity and reduced level of sedentary behavior were associated with psychological health among children and adolescents (15). Also, muscular strength among adolescents has been documented as an important health marker (28) and was shown to be associated with higher cardiometabolic risks (29). Making comparison between the two studies provide first-time evidence on the changes in the Jordanian adolescents’ lifestyle behaviors that have occurred over the course of these 14 years, and can be used towards planning, health policy decision-making and implementing adolescents’ health promotion programs in the country that aim toward enhancing healthy behaviors.

Therefore, the primary aim of the present paper was to describe the protocol, instrumentations, methodologies and implications of this comprehensive lifestyle investigation. This includes the collection of data on obesity, lifestyle behaviors (physical activity, sleep, and sedentary time), dietary habits, grip strength, and psychological well-being among Jordanian adolescents attending secondary schools in Amman city. An additional aim is to compare the levels of overweight/obesity, physical inactivity, sedentary time, dietary habits, and sleep duration with those data collected on Jordanian adolescents 14 years ago using the same sampling design and methodological procedures that were used in The Arab Teens Lifestyle Study (ATLS) (22, 23, 25, 26).

## 2 Study design and methods

### 2.1 Study design

This is a school-based cross-sectional study involving adolescents in secondary schools in Amman, Jordan. However, when comparing obesity and lifestyle behaviors between 2011 and 2025, the longitudinal design appears most appropriate and more robust to study the differences between the two periods but deemed impractical. Further, collecting data after 14 years from the same study participants would mean comparing data from them as adolescents in 2011 and now as adults leads to the use of different norms and standards.

### 2.2 Ethical approvals

Ethical approval was obtained from the Institutional Review Board at the University of Jordan in Amman, Jordan (#343/2024). A written informed consent will be acquired from all participating adolescents or from their parents if they are under 17 years of age. The research procedures will be conducted according to the principles expressed in the Declaration of Helsinki. Confidentiality of the data is ensured by coding them and keeping them in restricted access files. Approval of conducting this research in schools will be secured from the directorate of schools at the Ministry of Education in Amman and the principals of the selected schools.

The participants are not expected to experience harm in any way, be it emotional, physical or social, from any of the intended procedures of the present research. The participants will be informed that they could quit at any time if they felt not comfortable taking part in the study or are not willing to answer the questions.

### 2.3 Participant eligibility criteria

All Jordanian adolescents enrolled in secondary schools (grades 10–12) between the ages of 14 and 19 years in the city of Amman will be eligible for inclusion in the present study. Exceptions are if they have a medical condition that is not consistent with normal eating habits such as allergy to foods or eating disorders or having a medical reason that prevents the student from engaging freely in physical activity.

### 2.4 Sample size and sampling technique

A representative sample will be chosen using a multistage stratified cluster random sampling technique from male and female adolescents attending public and private secondary schools in East and West Amman city. The sample size is calculated using the following sample size equation for proportions: *n* = (z**^2^**pq) * D/d**^2^**) (30), while assuming a population proportion that yields the maximum possible sample size required (*P* = 0.50), with a confidence level of 95%, a margin of error (d) of 4%, and allowing for design effect (D) of 1.7, the sample size will be 1,020 participants. Adding nearly 30% for non-responses and missing data, the total sample size will be 1,320 participants. With this sample size, the statistical power while assuming a small effect size is 0.843.

Samples will be obtained from students enrolled in both public and private schools within west and east of Amman secondary schools. First, schools will be randomly selected and stratified according to gender, type of school (private and public), and location (west and east of Amman). In each gender, four schools will be randomly selected from each location (East and West) proportional to the number of students in public to private schools. The total number of randomly selected schools will be 16 from both girls’ and boys’ schools. Then, one class from each grade (10th, 11th, and 12th) will be randomly chosen from each selected school. Hence, a total of 24 classes will be surveyed in each of the girls’ and boys’ schools. All the students within these classes will be asked to voluntarily participate in the study. Then, will select randomly between 27 and 28 students from each class (the number of students in a class is usually around 36). This gives a selection rate of 75%. In case the number of students was less than the required one in a class, we will select another class from the same grade and complete the required number of participants. The total expected number of students will be 660 males and 660 females with a total sum of 1,320 students for both sexes. Table 1 presents the steps in sample selection procedures.

**Table 1:** Steps in sample selection from eastern and western Amman secondary schools.

Step	Eastern Amman Secondary Schools	Western Amman Secondary Schools	Total
1	A- Select randomly four schools from girls’ schools (from both public and private proportion to the number of students in each sector)	A- Select randomly four schools from girls’ schools (from both public and private proportion to the number of students in each sector)	16 schools
	B- Select randomly four schools from boys’ schools (from both public and private proportion to the number of students in each sector)	B- Select randomly four schools from boys’ schools (from both public and private proportion to the number of students in each sector)	
2	Select one class from grades 10, 11, & 12 (total number of classes = 24)	Select one class from grades 10, 11, & 12 (total number of classes = 24)	48 classes
3	Select randomly between 27 and 28 students from each class (if response rate in one class is low, select another class to complete the number of selected students)	Select randomly between 27 and 28 students from each class (if response rate in one class is low, select another class to complete the number of selected students)	1,320 students
4	Number of students = 660 (330 males)	Number of students = 660 (330 males)	

### 2.5 Measurement procedures

Table 2 illustrates all variables that are going to be measured in the current research as well as their methods of assessment.

**Table 2:** Variables assessed in the present research. .

Assessed variable and measurement units	Assessment method
Anthropometric measurements (weight (kg), height (cm), body mass index (kg/m**^2^**), waist circumference (cm), waist height ratio (%)	Direct measurement or computed using established equations
Demographics and socioeconomic status [age (years), paternal and maternal education levels, monthly family income (Jordanian Dinar), GPA]	Self-reported
Lifestyle behaviors:	
Physical activity (min/week; METs-min/week)	Self-reported (validated questionnaire)
Sedentary behaviors (hours/day)	Self-reported (developed and pre-tested questionnaire)
Sleep duration (hours/night)	Self-reported
Dietary habits (frequency/week)	Self-reported (validated questionnaire)
Psychological Well-being (scores 10 to 50)	Self-reported (validated questionnaire)
Grip strength (kg; Kg/kg of body weight; kg/height)	Direct measurement by hand dynamometer

The following description provides more details on the assessed variables.

#### 2.5.1 Anthropometric measurements

Anthropometric variables include body weight (kg) and height (cm) as well as the calculated body mass index (BMI). Measurements will be performed in the morning by a trained researcher according to standardized procedures. A student's privacy is going to be taken into consideration when taking all anthropometric measurements. Body weight will be measured to the nearest 100 grams using calibrated portable medical scales (manufactured in China). All measurements will be done with minimal clothing and without shoes. Height will be measured to the nearest centimeter while the subject is in a full standing position without shoes using a calibrated measuring rod with graduated measuring tape fixed to it (locally made). BMI is going to be calculated as the ratio of weight in kilograms by the height squared in meters. The International Obesity Task Force (IOTF) age- and sex-specific BMI cutoff reference standards (31) are going to be used to identify overweight and obesity among adolescents between the ages of 14 and 17 years. For participants 18 years and older, we will use WHO adult cutoff points of 25–29.9 kg/m**^2^** to define overweight and 30 kg/m**^2^** and higher for obesity. In addition, waist circumference (WC) will be measured horizontally at one cm below navel level and at the end of gentle expiration to the nearest 0.1 cm using a non-stretchable measuring tape. Waist height ratio (WHtR) is going to be calculated as the ratio between WC in cm and height in cm. A WHtR cut-off point of 0.50 is used to define abdominal obesity in males and females (32, 33).

#### 2.5.2 Demographics and socioeconomic status

In addition to age in years, we will report important demographics and socioeconomic status of the participating adolescents. These include educational levels of both parents as well as the family monthly income in Jordanian Dinar (JD = 1.4 US Dollar). Monthly income is categorized into 5 categories (less than 1000 JD, 1001-2000 JD, 2001-3000 JD, 3001-4000 JD, and above 4000 JD). Economic status has been found to be an important moderator of the association between parent-child physical activity and screen time in adolescents (34). Also, recent research showed that a positive association was found between Korean adolescents’ physical activity and socioeconomic indicators (35). Additionally, we assessed the number of children under the age of 18 living in the home.

#### 2.5.3 Assessments of lifestyle behaviors

Assessment of physical activity, sedentary behaviors, and dietary habits are going to be through self-reported validated questionnaires, as shown in Table 2. A detailed description of the procedures is as follows.

##### 2.5.3.1 Physical activity

The Arab Teen Lifestyle Study (ATLS) questionnaire is going to be used to collect lifestyle variables (22, 23). The questionnaire was previously shown to be valid and reliable for assessing physical activity and other lifestyle habits in youth from 14 to 25 years of age (22, 23, 36). The questionnaire collects information on the frequency, duration and intensity of light-, moderate- and vigorous-intensity physical activities during a typical week. The questionnaire covers several domains of activity including transport, household, fitness and sporting and leisure-time activities. The total time spent in minutes on all activity per week as well as time spent in moderate- and vigorous-intensity physical activity are used as indicators of physical activity levels. In addition, we will ask the students about the ways they travel to and from schools.

Furthermore, to determine the participants’ levels of physical activity, we will use the total activity energy expenditure in metabolic equivalent of task (MET) in minutes per week and the METs-minutes per week spent in each of the moderate- and vigorous-intensity physical activity. The MET values correspond to physical activity intensity according to adults’ and youths’ compendium of physical activity (37, 38). To calculate the percentage of adolescents who meet the daily physical-activity recommendations, we will use two cutoff scores equivalent to one hour per day of moderate-intensity (4 METs) physical activity or one hour per day of moderate- to vigorous-intensity (6 METs) physical activity (22, 23). We then converted these two exercise intensities (Moderate and moderate to vigorous physical activity) into activity energy expenditure in METs-min per week, thus, corresponding to 1680 METs-min/week (60 min per day  ×  7 days per week  ×  4 METs) and 2520 METs-min per week (60 min per day × 7 days per week  ×  6 METs). Any participant having 420 min per week of at least moderate physical activity will have achieved sufficient physical activity (6). The prevalence of sufficiently vs. insufficiently active participants will be compared.

##### 2.5.3.2 Sedentary behaviors

Questions on sedentary behaviors are part of the ATLS questionnaire. They are intended to determine important information from the parents about the typical daily time spent on sedentary activities (screen time), including time spent viewing TV, playing video games, and computer and Internet recreational use. Participants are expected to provide the average number of daily hours during each of weekdays and weekends that are spent on total screen time. For the sedentary time cutoff hours, we will use the American Academy of Pediatrics guidelines of a maximum of 2 h per day (39). In addition, if the proportion of screen time is high (if 80% of the sample having 2 h or more of screen time per day), we will use a cutoff score of equal or above 3 h of screen time per day.

In addition, we are going to use an Arabic version of Sedentary Behavior Questionnaire (ASBQ), which was recently developed for assessing sedentary sitting time during weekdays and weekends for youth and adults (40). It is a more extensive multidimensional sedentary behaviors questionnaire with 13 items covering a wide range of sedentary activities. The ASBQ exhibited excellent content validity for assessing sedentary time in adolescents and adults with a very high item-level and scale-level content validity index. Its kappa statistic, a measure of interrater reliability, was 0.95 (40).

##### 2.5.3.3 Sleep duration

Sleep duration during each weekday (school days) and weekends will be assessed by the same ATLS questionnaire. We defined insufficient sleep (short sleepers) as sleeping less than 8 h per night, according to the definition of the National Sleep Foundation for school-age children 14–17 years and less than 7 h per night for those 18–19 years of age (41).

##### 2.5.3.4 Dietary habits

The ATLS questionnaires (22) included 10 specific questions related to the frequency of consuming certain dietary habits during a typical (usual) week. The questions asked the participants to state how many times per week they consume breakfast, vegetables (cooked and uncooked), fruits, milk and dairy products, sugar-sweetened drinks (including soft drinks), fast foods, donuts/cakes, sweets and chocolates and energy drinks. The questions covered both healthy and unhealthy dietary habits. The adolescent is given a choice of answers, ranging from zero day per week (never) to a maximum intake of 7 days per week (every day). The dietary frequency questionnaire that is part of ATLS had been previously validated (42). We will use cut-off values of daily vs. non-daily intakes for healthy dietary habits consumption, such as breakfast, vegetables, fruit, and milk/dairy products. As for the unhealthy dietary intakes, such as sweetened drinks, fast foods, French fries/potato chips, cake/donuts, and chocolates/candy, the cut-off values will be intakes of less than 4 days per week vs. 4 or more intakes per week (22–24).

#### 2.5.4 Assessing psychological well-being

The Brief Psychological Well-being Scale (BPWBS) will be used to assess psychological health (43). It is a short version of the comprehensive psychological well-being scale. It measures various aspects of good psychological functioning such as relationships and mastery. Brief BPWBS contains 10 items. Response can be rated on a 5-point scale ranging from strongly disagree to strongly agree (scores from 1 to 5). Scores range from 10 to 50. Scoring categories represent five classes: very low (scores 10-30); low (scores 31–37); average (scores 38–40); high (scores 41–44); and very high (scores 45–50). BPWBS was translated to and validated in Arabic language by using standard procedure of back translation (43). Psychometric properties of the Arabic translated version of the BPWBS were investigated using Cronbach's alpha (inter consistency = 0.91) and by confirmatory factor analysis (construct validity = 0.947) as well as content validity (43).

#### 2.5.5 Measurement of grip strength

Grip strength represents a measure of upper body muscular strength. Muscle strength has been recognized as an important health marker in adolescents and strong inverse associations were shown between muscular fitness and total and central adiposity, as well as with metabolic and cardiovascular risks (28). Also, absolute grip strength as well as that normalized for height were related to higher cardiometabolic risk factors among adolescent males (29). In addition, handgrip strength was shown to be associated with insulin resistance and glucose metabolism among adolescents (44). By including a measure of muscle strength among adolescents in the present study, we can examine the interrelationships between obesity and health behaviors. Grip strength is going to be measured using a hand-held digital dynamometer (Takei, Japan). In the present research, we are going to use absolute grip strength, normalized to body weight, and normalized to BMI or height square (45). The adolescent grabs the dynamometer in his dominant arm and flexes his/her grip as maximal as possible, while in standing position and the arm is not touching the thigh. Participant is given three attempts, and the best one is recorded.

### 2.6 Pilot study

A sample of 25 male and female students from various public and private schools in Amman served as participants for the pilot work. The purpose of the pilot study was to check on the best management approach to data collection and to find out how much time it takes to collect the data from the adolescents. It also served to train the research assistants in the procedures and management of data collection. Data collection included conducting anthropometric measurements and answers to the three types of questionnaires (lifestyle questionnaire, sedentary behaviors questionnaire, and psychological well-being questionnaire). The adolescents efficiently responded to the questionnaires and socioeconomic variables using electronic forms. All data collection took 25 min. In addition, the procedures showed smooth experiences by the responding students. One research assistant under the supervision of a researcher will assume and conduct one task during assessment of the study's variables. This is to eliminate the inter-rater differences in measurements.

### 2.7 Data and statistical analysis

Data will be entered into an SPSS data file, checked, cleaned and analyzed using the IBM SPSS Statistics for Windows, version 28 (IBM Corp., Armonk, N.Y., USA). To avoid over-reporting, physical-activity scores will be cleaned and truncated at reasonable and realistic levels, considering the long time spent in learning at schools, doing homework, sleeping and screen time. The maximum total time spent on physical activity per week is going to be truncated to 28 h or 4 h of physical activity per day according to previous studies (23, 46, 47). In addition, the maximum number of stair levels taken by students per day is capped to 30 floors. Total screen time (combined time of TV viewing, computer use and Internet time) is going to be truncated at 16 h per day (23, 46, 47).

Descriptive data from participants will be summarized separately for boys and girls. Continuous variables will be tested for normality by established methods. Depending on the expected data assumptions like normality, linearity, independence, and equality of variance, the appropriate statistical tests will be carried out. The normally distributed variables will be presented as frequencies, means, standard deviation or standard error. In cases of severe non-normal distribution, we will apply the equivalent appropriate non-parametric tests of significance and report median and interquartile range values. The approach for missing data would depend on the amount of the missing data, but generally starts with preventive measures, followed by careful imputation for low systematic absence of data in a dataset or deletion for small amounts of random missing data.

The prevalence of sufficiently and insufficiently active adolescents will be calculated. Pearson's correlation analysis will also be conducted between selected variables. Differences between breakfast skippers and non-skippers, boys and girls, public vs. private schools, active and inactive participants, low vs. high screen time, sufficient vs. insufficient sleepers and participants with overweight/obesity and without overweight/obesity will be compared using the classical statistical tests of significance (t-tests and ANOVA) with adjustment to confounders. Bonferroni correction test will be used for multiple comparisons. Additionally, we will perform a Multivariate Analysis of Covariance (MANCOVA) to compare the sample means of a set of two or more dependent continuous variables (such as lifestyle behaviors) across groups of independent categorical variables, while controlling for age or sociodemographic factors. It increases the ability to identify differences by removing the influence of covariates from the error variance. MANCOVA determines if group means differ across a set of related outcomes, thus reducing Type I error. To study the quantitative effects of different independent variables on adolescent adiposity, sedentary behaviors, sleep duration, demographics and SES, etc. (dependent outcome variables), we will use logistic regression analysis and other multivariable statistical methods. When there is clustering effect, as indicated by the intracluster correlation coefficient (ICC) for the primary outcome, multilevel mixed-effects logistic regression is going to be employed to assess the associations between lifestyle behaviors, in which the log odds of the outcomes are modeled as a linear combination of the predictor variables when data are clustered or there are both fixed and random effects. Pearson's correlation and linear regression will be used for prediction purposes when appropriate. The level of significance will be set at a value of ≤ 0.05.

## 3 Discussion

The aim of the current study was to describe the protocol, instrumentations, methodologies and implications of this comprehensive lifestyle behavioral investigation. This includes the collection of data on obesity, lifestyle behaviors (physical activity, sleep, and sedentary time), dietary habits, grip strength, and psychological well-being among Jordanian adolescents attending secondary schools in Amman city. To our knowledge, the present research is the first study to cross-sectionally revisit the obesity and lifestyle behaviors of Jordanian adolescents using the same protocol and methodologies as the previous study that was conducted in 2011. The Jordanian peoples though considered part of the Arabic community in the Middle East, findings from research conducted earlier using ATLS 2011 (25, 26) indicated that their obesity level was much lower than those of adolescents in neighboring countries like Saudi Arabia (23) or Kuwait (48). A considerable proportion of Jordanian adolescents exhibited undesirable lifestyle habits (26). Also, they displayed somewhat varying lifestyle behaviors compared to other countries (25, 26). The weather in Jordan is also milder than Saudi Arabia or Kuwait where temperature during the summer months can exceed 44 degrees Celsius, thus discouraging outdoor physical activity in those two countries. The present research should better highlight what particular contributions this cross-sectional follow-up study in Jordan will make relative to other ATLS-related projects in the region (e.g., Saudi Arabia) (46, 47).

The current research will provide us with an opportunity to collect, analyze, and report important lifestyle information from Jordanian adolescents using standardized data collection procedures like the ones used in previous study conducted 14 years ago in Amman, Jordan. In addition, the current research will enable us to gain insight and understanding of the participants’ facilitators and barriers to physical activity among Jordanian adolescents and how such factors changed over time since they were previously examined in 2011. It also provides perspectives on the interactions of obesity, socioeconomic status, grip strength, and psychological wellbeing with lifestyle-related outcomes such as physical activity, sedentary behaviors, sleep, and dietary habits.

In the current study, we are assessing the same previous parameters that were tested in 2011 study, such as overweight or obesity, physical activity, sedentary behaviors, sleep, and dietary habits. In addition, we will examine new variables such as grip strength, GPA, trips to and from schools, and psychological health and wellbeing of the Jordanian adolescents. The later variables will be tested for the first time in the current project and expect to add more insights and perspectives into the relationships between psychological health and lifestyle behaviors among Jordanian adolescents.

It is well recognized that continuous tracking of lifestyle behaviors is crucial to identify risk-groups with physical inactivity, insufficient sleep, increased screen viewing time, and unfavorable dietary habits. This is important information as a prerequisite to promote healthy lifestyle behaviors and guide future interventions. It has been well acknowledged that continued research on the lifestyle behaviors of Jordanian youth is important as diet, sleep, sedentary behaviors, and physical activity play important roles in maintaining health and preventing diseases (49). In fact, the WHO has recently released a landmark document highlighting the importance of increasing physical activity and decreasing sedentary time for all ages, including children and adolescents (6). Moreover, as apart of the Global Strategy for Women's, Children's and Adolescents’ Health (2016-2030), the WHO identified institutions and policies as fundamental components for promoting a good nutritional status in preventing excess body weight for people from all countries (50).

Worldwide, childhood obesity is a significant public health concern, and the prevalence of pediatric obesity has considerably increased across developed and developing countries (51). In Jordan, overweight and obesity amongst Jordanian adolescents (15–18-year-olds) reached 22.4% according to a study findings published in 2016 (19). Based on data from the Jordanian Department of Statistics, 44.3% of the population of Jordan is under 20 years of age (5.1 million of children and adolescents) (52). It is estimated that the research findings will provide cross-sectional, yet important information related to trends occurring in overweight/obesity and lifestyle behaviors among Jordanian adolescents over the last 14-year period. The results will also provide more understanding of the lifestyle-related determinants and associations of obesity among Jordanian adolescents. This will have important implications for planning and executing preventive measures and promotional programs toward the health and prosperity of adolescents with excess weight problems (53).

Our sample will be drawn from a representative sample from public and private schools in the city of Amman. Amman is the capital and the largest city in Jordan with a cosmopolitan population. The school setting is well suited for large-scale physical activity initiatives, especially when almost all adolescents enrolled in schools in Jordan. Information gathered from the current study will most likely pave the way for more focused future interventions aiming towards promoting lifestyle behaviors and wellbeing of children and adolescents in Jordan. Such approach is likely to have a positive impact not only on the health and wellbeing of Jordanian adolescents, but also on their cognitive and academic performance, as indicated by studies published elsewhere (54–56).

## 4 Significance of study

Adolescent obesity is arguably the most serious public health challenge of the present century (57) and its prevalence among Jordanian children and adolescents is rising over the last decades (19, 20, 58). During these years, adolescents have become more independent and have increased access to food choices apart from those available at home. It is also in this period that adolescents increase their social interactions with peers of similar age and develop individual eating habits and physical-activity patterns. Dietary habits appear to be established in the mid-teens and were shown to be closely related to lifestyle (10, 11). A better understanding of the relationships between healthy behaviors and psychological well-being status among young people is considered necessary for effective prevention and management of lifestyle-related risk factors. Some reports have even suggested that diet, physical activity and sedentary behaviors should be considered simultaneously in the prevention of cardio-metabolic disease (14).

It is essential to examine and monitor the reciprocal flow of lifestyle factors that influence overweight and obesity during adolescence. At present, there is a real concern about the escalating trend of unhealthy lifestyle behaviors by youth, including inactivity, sedentary habits, insufficient sleep, and skipping breakfast with increased consumption of sweetened soft drinks, and the possible role of these habits in the pathogenesis of childhood obesity (59). Consequently, it is anticipated that the findings of the present research will provide valuable and updated data on obesity status, lifestyle behaviors, dietary habits, and psychological well-being of Jordanian adolescents living in Amman. The findings of this research are expected to have some implications for the health and well-being of Jordanian adolescents and can be used effectively for planning and implementing adolescent's future health promotion programs in Jordan.

## 5 Strengths and limitations

The present study has several strengths. First, we are collecting a large volume of data related to many variables of interest, which provides a bigger picture of exposures and outcomes and helps in explaining the interrelationships between the variables under investigation and their confounders. Second, it uses the same instruments, design, and methodology as the previous study conducted in 2011. Therefore, comparisons with past findings can be made without due limitations. Third, inclusion of waist circumference as a measure of central obesity, in addition to BMI, will further enhance our understanding of the behavioral correlates of Jordanian adolescents, especially since it is known that waist circumference and waist to height ratio are better predictors of cardiovascular disease in children than BMI alone (60). Fourth, the feasibility study that was conducted in the current research is expected to optimize the protocol and design of the study for successful implementation of the data collection in the school settings. An additional strength is that we are collecting more variables this time compared with the previous study 14 years ago. This will enhance our understanding when examining the associations between obesity and lifestyle behaviors and physical and psychological wellbeing of young Jordanian people.

As to the limitations of the current research, the cross-sectional design of the present study, which cannot imply any causality, is a potential limitation. Also, when comparing obesity and lifestyle behaviors between 2011 and 2025, the longitudinal design appears most appropriate and more robust to study the differences between the two periods but deemed impractical. Nevertheless, there are still significant contributions that can be achieved by cross-sectional research as they can lead the way to more prospective studies. Further, despite using a well-described and validated lifestyle and psychological wellbeing instruments in the present study, they are still considered as subjective tools that have their inherent limitations. Self-reported questionnaires suffer sometimes from potential for recall bias and social desirability effects. However, the instruments have been shown to be valid and reliable (22, 36, 61) and can provide much information about the types of physical activities, barriers and facilitators to active living compared with objective instruments. The social and environmental transformations over the 14-year period and the emergence of digital technology may have an impact on lifestyle behaviors among the Jordanian adolescents. Also, the assessment of adolescents’ dietary behaviors in the present study is self-reported. However, the instrument has been extensively used in previous studies among adolescents and youths in different countries and has proven to be useful and reliable (23, 25, 47, 48). Besides that, to compare the findings from the study conducted in Amman in 2011 with the findings from the current study, we need to have used the same instruments. Additionally, the study included youth from the city of Amman, the capital of Jordan and although Amman is a cosmopolitan city, the findings of the present study cannot be generalized to youth in rural Jordan, who may have different lifestyle behaviors.

## Data Availability

The original contributions presented in the study are included in the article/Supplementary Material, further inquiries can be directed to the corresponding author.
