# Supplementary material for: A methodological study revisiting obesity and lifestyle behaviors of Jordanian adolescents in Amman after 14 years
Source: Front Sports Act Living. 2026 Jun 17;8:1841128. doi: 10.3389/fspor.2026.1841128 (PMC13318693; doi:10.3389/fspor.2026.1841128)
Supplement: Supplementary file 1 [file Datasheet1.pdf]

Arab Teens Lifestyle (ATLS) Questionnaire (Revised 2018)

Dear participant,

The purpose of this questionnaire is to assess your lifestyle habit, including physical activity, sedentary behaviors and dietary habits. Therefore, we would like you to answer the following questions as accurately as possible by ticking the box that represents your choice. What we are asking for is your activity (behaviors) in a typical (usual) week. All information provided will be treated with strict confidentiality and used only for research purposes.

Name (optional): …………………………………………… **Date:** ……………………

---

1) School name: …………………………………… **2- District:** ……………………...

3) Study Level (grade): ………….

4) Age (in years) …… **5) Weight (kg)** ……

6) Height (cm) …... **7) Waist circumference (cm)** …...

PART ONE: *Physical Activity/Inactivity*

8) How many <u>days</u> per week do you regularly walk?

None ☐ 3 days ☐ 6 days ☐

One day ☐ 4 days ☐ 7 days (daily) ☐

2 days ☐ 5 days ☐

9) If you regularly walk, what is the pace of your walk?

Slow ☐

Moderate ☐

Fast ☐

10) If you regularly walk, how many minutes do you walk each day?

Number of minutes: …….

11) How many times per DAY you use the stairs in school, home, or elsewhere? (one floor of stair counts as 1 time)

None ☐ 3 times ☐ More than 5 ☐

Once ☐ 4 times ☐ *If more than 5 times, how many? …….……..*

Twice ☐ 5 times ☐

12) How many <u>days</u> per week do you regularly jog or run?

None ☐ 3 days ☐ 6 days ☐

One day ☐ 4 days ☐ 7 days (daily) ☐

2 days ☐ 5 days ☐

13) If you regularly jog or run, how many minutes do you do each day?

Number of minutes: ……

14) How many <u>days</u> per week do you regularly cycle (either on an outdoor or a stationary cycle)?

None ☐ 3 days ☐ 6 days ☐

One day ☐ 4 days ☐ 7 days (daily) ☐

2 Days ☐ 5 days ☐

15) If you use an outdoor or a stationary cycle regularly, how many minutes do you cycle each day?

Number of minutes: ……

16) How many days per week do you regularly swim?

None	☐	3 days	☐	6 days	☐
One day	☐	4 days	☐	7 days (daily)	☐
2 days	☐	5 days	☐		

17) If you regularly swim, how many minutes do you swim each day?

Number of minutes ……

18) How many times per week do you regularly engage in moderate intensity sports (e.g. volleyball, table tennis, bowling, badminton, aerobic dance or other similar activities)?

None	☐	3 times	☐	6 times	☐
Once	☐	4 times	☐	7 times or more	☐
Twice	☐	5 times	☐		

19) If you regularly play moderate intensity sports, how many minutes do you play each time?

Number of minutes: ……

20) How many times per week do you regularly engage in high intensity sports (e.g. soccer, rugby, hockey, netball, basketball, handball, athletics, tennis, squash, rope jump, cross fit, etc.)?

None	☐	3 times	☐	6 times	☐
Once	☐	4 times	☐	7 times or more	☐
Twice	☐	5 times	☐		

21) If you regularly play high intensity sports, how many minutes do you play each time?

Number of minutes: ……

22) How many times per week do you participate in self-defence sports (e.g. kick-boxing, judo, karate, taekwondo, etc.)?

None	☐	3 times	☐	6 times	☐
Once	☐	4 times	☐	7 times or more	☐
Twice	☐	5 times	☐		

23) If you participate in self-defence sports regularly, how many minutes do you do each time?

Number of minutes: ……

24) How many times per week do you regularly do strength training (weight training or body building or calisthenics exercise)?

None	☐	3 times	☐	6 times	☐
Once	☐	4 times	☐	7 times or more	☐
Twice	☐	5 times	☐		

25) If you regularly do strength training (weight training, body building or calisthenics exercise), how many minutes do you do it each time?

Number of minutes: ……

26) How many times per week do you engage in household work (e.g. gardening, vacuuming, washing, car cleaning)?

None	☐	3 times	☐	6 times	☐
Once	☐	4 times	☐	7 times or more	☐
Twice	☐	5 times	☐		

27) If you do household work, how many minutes does it take per day?

Number of minutes: ……

28) How many times per week do you do traditional dancing (whether alone or with your friends)? (*__This question is specifically for girls, and boys can go directly to item 30__*).

None	☐	3 times	☐	6 times	☐
Once	☐	4 times	☐	7 times or more	☐
Twice	☐	5 times	☐		

29) If you do dancing, how many minutes do you do such activities each time?

Number of minutes: ……

30) Where do you normally do your physical activities or sports?

Home	☐	Park or public area	☐	Health/Fitness Club	☐
School	☐	Sports or recreation centre	☐	Other places *Please give details* …………….…………..	☐

31) With whom do you normally do your physical activities or sports?

Alone	☐	School peers	☐	Other relatives	☐
Friends	☐	Parents	☐	Other *Please give details* …………….…………	☐

32) When do you usually do your physical activities or sports?

Morning	☐	Afternoon	☐	After evening meal	☐
Noon time	☐	Evening	☐	No specific time	☐

33) If you __participate__ in physical activities or sports regularly, what are the main reasons for that? *__Please answer either item 33 (if you are active) or item 34 (if not active__*).

Health	☐	Social	☐	Competition	☐
To lose weight	☐	Recreation	☐	Others *(please give details)* …………………………	☐

34) If you __don't__ participate in physical activities or sports regularly, what are the main reasons for that?

No time	☐	No suitable facilities	☐	Afraid of criticism	☐
Not important	☐	Health reasons	☐	Others *(please give details)* …………………………..	☐

PART TWO: *Sedentary Behaviors*

35) On average, how long __per day__ do you watch TV and/or DVD/Video, or use computer and/or the internet for leisure including social media during __weekdays__?

I do not watch TV	☐	2 hours	☐	5 hours	☐
½ hour	☐	3 hours	☐	6 hours	☐
1 hours	☐	4 hours	☐	*If more than 6 hours, how many?*	

36) On average, how long per day do you watch TV and/or DVD/Video, or use computer and/or the internet for leisure including social media during weekends?

I do not watch TV	☐	2 hours	☐	5 hours	☐
½ hour	☐	3 hours	☐	6 hours	☐
1 hours	☐	4 hours	☐	*If more than 6 hours, how many?*	

37) On average, how many hours per day do you sleep during weekdays?

3 hours	☐	6 hours	☐	9 hours	☐
4 hours	☐	7 hours	☐	10 hours or more	☐
5 hours	☐	8 hours	☐		

38) On average, how many hours per day do you sleep during weekends?

3 hours	☐	6 hours	☐	9 hours	☐
4 hours	☐	7 hours	☐	10 hours or more	☐
5 hours	☐	8 hours	☐		

PART THREE: *Dietary Habits*

39) How many times (days) per week do you have your breakfast?

I don’t have breakfast	☐	3 times	☐	6 times	☐
Once	☐	4 times	☐	7 times	☐
Twice	☐	5 times	☐		

40) How many times per week do you drink sugary drinks /soft drinks (e.g. Coke, Pepsi, 7up, Sports drink)?

None	☐	3 times	☐	6 times	☐
Once	☐	4 times	☐	7 times or more	☐
Twice	☐	5 times	☐		

41) How many times per week do you eat vegetables (fresh or cooked)?

None	☐	3 times	☐	6 times	☐
Once	☐	4 times	☐	7 times or more	☐
Twice	☐	5 times	☐		

42) How many times per week do you eat fresh fruit?

None	☐	3 times	☐	6 times	☐
Once	☐	4 times	☐	7 times or more	☐
Twice	☐	5 times	☐		

43) How many times per week do you have dairy products (e.g. milk, yogurt, cheese)?

None	☐	3 times	☐	6 times	☐
Once	☐	4 times	☐	7 times or more	☐
Twice	☐	5 times	☐		

44) How many times per week do you eat fast food (e.g. burgers, sausage, pizza, or Arabic shawarma, inside or outside your home?

None ☐ | 3 times ☐ | 6 times ☐
Once ☐ | 4 times ☐ | 7 times or more ☐
Twice ☐ | 5 times ☐

45) How many times per week do you eat French fries and/or potato chips?

None ☐ | 3 times ☐ | 6 times ☐
Once ☐ | 4 times ☐ | 7 times or more ☐
Twice ☐ | 5 times ☐

46) How many times per week do you eat cakes, biscuits, donuts, or similar food?

None ☐ | 3 times ☐ | 6 times ☐
Once ☐ | 4 times ☐ | 7 times or more ☐
Twice ☐ | 5 times ☐

47) How many times per week do you eat sweets and/or chocolates?

None ☐ | 3 times ☐ | 6 times ☐
Once ☐ | 4 times ☐ | 7 times or more ☐
Twice ☐ | 5 times ☐

48) How many times per week do you drink energy drinks (e.g. Red Bull, Power Horse)?

None ☐ | 3 times ☐ | 6 times ☐
Once ☐ | 4 times ☐ | 7 times or more ☐
Twice ☐ | 5 times ☐

Thank you for completing this questionnaire and taking part in this research.
