# Supplementary material for: A methodological study revisiting obesity and lifestyle behaviors of Jordanian adolescents in Amman after 14 years
Source: Front Sports Act Living. 2026 Jun 17;8:1841128. doi: 10.3389/fspor.2026.1841128 (PMC13318693; doi:10.3389/fspor.2026.1841128)
Supplement: Supplementary file 2 [file Datasheet2.pdf]

Sedentary Behavior Questionnaire (SBQ)

Sedentary behaviors are any waking behavior that involves sitting, lying or reclining position apart from sleep. When asked about sedentary behaviors, you must think about behaviors that meet these characteristics.

Section 1:

- **During SCHOOL HOURS**, How many hours per day do you spend sitting **during school hours**? Respond thinking about the **average** time you spend during a typical (usual) week.

1. How many **days per week** do you typically **go to** school?	[ ] days
2. How many **hours per day do you spend** sitting while at school?	[ ] hrs. [ ] min

Section 2:

- **How long have you been engaged in the following behaviors per day during weekdays or weekends?**

Respond to the following questions thinking about AVERAGE time you spend on each activity during a typical (usual) weekdays and weekends. **Each period of sitting down should only be entered once**. For example, if you spent one hour sitting on the sofa reading a book while you were listening to music, count this time as one hour reading if this was your main activity. Do not count this as one hour reading a book and another hour listening to music.

**How many** hours/min per **day** do you spend **sitting, lying or reclining while:**	**During weekdays**	**During weekends**
3. Watching movies, TV, videos (regardless of the source-TV, computer, phone)	[ ] hrs. [ ] min	[ ] hrs. [ ] min
4. Playing computer/video games	[ ] hrs. [ ] min	[ ] hrs. [ ] min
5. Internet surfing or using social media for fun	[ ] hrs. [ ] min	[ ] hrs. [ ] min
6. Doing homework/studying	[ ] hrs. [ ] min	[ ] hrs. [ ] min
7. Reading for fun	[ ] hrs. [ ] min	[ ] hrs. [ ] min
8. Sitting and talking with family or friends in person or via internet	[ ] hrs. [ ] min	[ ] hrs. [ ] min
9. Listening to Quran, Radio, or music (without doing anything else)	[ ] hrs. [ ] min	[ ] hrs. [ ] min
10. Resting (lying down, but not taking a nap, etc.)	[ ] hrs. [ ] min	[ ] hrs. [ ] min
11. Doing hobbies that require thinking/reasoning (doing puzzles, playing cards, doing crossword puzzles, etc.)	[ ] hrs. [ ] min	[ ] hrs. [ ] min
12. Doing crafts or art work <u>while sitting</u> (like drawing, knitting, sewing, etc.)	[ ] hrs. [ ] min	[ ] hrs. [ ] min
13. Using transportation while sitting (in car, bus, train, subway or motorbike)	[ ] hrs. [ ] min	[ ] hrs. [ ] min
14. Doing household tasks <u>while seated</u> (cooking, ironing, slicing foods, etc.)	[ ] hrs. [ ] min	[ ] hrs. [ ] min
15. Engaged in other activities than the above <u>while seated</u>, name them: ………………………………………………………………………………………………	[ ] hrs. [ ] min	[ ] hrs. [ ] min

16. Typically, how often do you **interrupt your sitting** time during a typical day in a week?				
□ every 30 min or less	□ every 1 hour	□ every 2 hours	□ every 3 hours	□ every 4 hours or more

Thank you for participating in this survey.
