# Supplementary material for: A methodological study revisiting obesity and lifestyle behaviors of Jordanian adolescents in Amman after 14 years
Source: Front Sports Act Living. 2026 Jun 17;8:1841128. doi: 10.3389/fspor.2026.1841128 (PMC13318693; doi:10.3389/fspor.2026.1841128)
Supplement: Supplementary file 3 [file Datasheet3.pdf]

Psychological Weel-being Questionnaire

Kindly, place (√) sign in front of your answer to the following questions:

Item	I strongly disagree (1)	I do not agree (2)	Neutral (3)	I agree (4)	I strongly agree (5)
1- I have a clear goal and meaning of my life					
2- I am optimistic toward my future					
3- My life is running good					
4- I feel good most of time					
5- What I am doing in my life has value and meaning					
6- I could succeed if I decided so					
7- I am right now attaining most of my goals					
8- I feel vigor and vitality in most activities that I do					
9- There are people who appreciate me for my self					
10- I feel how important it is to belong to my society					
